# Targeting PELP1 oncogenic signaling in TNBC with the small molecule inhibitor SMIP34

**DOI:** 10.1007/s10549-023-06958-4

**Published:** 2023-05-18

**Authors:** Kristin A. Altwegg, Uday P. Pratap, Zexuan Liu, Junhao Liu, John R. Sanchez, Xue Yang, Behnam Ebrahimi, Durga Meenakshi Panneerdoss, Xiaonan Li, Gangadhara R. Sareddy, Suryavathi Viswanadhapalli, Manjeet K. Rao, Ratna K. Vadlamudi

**Affiliations:** 1 Department of Obstetrics and Gynecology, University of Texas Health San Antonio, San Antonio, TX 78229 USA; 2 Department of Oncology, Xiangya Hospital, Central South University, Changsha, 410008 Hunan People’s Republic of China; 3 Department of Obstetrics and Gynecology, Second Xiangya Hospital, Central South University, Changsha, 410011 Hunan People’s Republic of China; 4 Greehey Children’s Cancer Research Institute, University of Texas Health San Antonio, San Antonio, TX 78229 USA; 5 Mays Cancer Center, University of Texas Health San Antonio, San Antonio, TX 78229 USA; 6 Audie L. Murphy Division, South Texas Veterans Health Care System, San Antonio, TX 78229 USA

**Keywords:** PELP1, SMIP34, TNBC, Ribosome, Rix complex

## Abstract

**Purpose:**

Triple-negative breast cancer (TNBC) is the most aggressive subtype of breast cancer. Oncogenic PELP1 is frequently overexpressed in TNBC, and it has been demonstrated that PELP1 signaling is essential for TNBC progression. The therapeutic utility of targeting PELP1 in TNBC, however, remains unknown. In this study, we investigated the effectiveness of SMIP34, a recently developed PELP1 inhibitor for the treatment of TNBC.

**Methods:**

To ascertain the impact of SMIP34 treatment, we used seven different TNBC models for testing cell viability, colony formation, invasion, apoptosis, and cell cycle analysis. Western blotting and RT-qPCR were used to determine the mechanistic insights of SMIP34 action. Using xenograft and PDX tumors, the ability of SMIP34 in suppressing proliferation was examined both ex vivo and in vivo.

**Results:**

TNBC cells’ viability, colony formation, and invasiveness were all decreased by SMIP34 in in vitro cell-based assays, while apoptosis was increased. SMIP34 treatment promoted the degradation of PELP1 through the proteasome pathway. RT-qPCR analyses confirmed that SMIP34 treatment downregulated PELP1 target genes. Further, SMIP34 treatment substantially downregulated PELP1 mediated extranuclear signaling including ERK, mTOR, S6 and 4EBP1. Mechanistic studies confirmed downregulation of PELP1 mediated ribosomal biogenesis functions including downregulation of cMyc and Rix complex proteins LAS1L, TEX-10, and SENP3. The proliferation of TNBC tumor tissues was decreased in explant experiments by SMIP34. Additionally, SMIP34 treatment markedly decreased tumor progression in both TNBC xenograft and PDX models.

**Conclusions:**

Together, these findings from in vitro, ex vivo, and in vivo models show that SMIP34 may be a useful therapeutic agent for inhibiting PELP1 signaling in TNBC.

## Background

Globally, breast cancer (BC) accounts for 1 in 4 cancer cases and 1 in 6 cancer deaths, ranking first for incidence in many countries and responsible for 685,000 deaths annually (6.9% of cancer deaths) [1]. Among the many BC subtypes, estrogen receptor-positive (ER+) breast cancer accounts for 60–70% of cases, HER2 overexpression accounts for 10–15%, and triple negative breast cancer (TNBC) accounts for 15–20% of cases [2]. TNBC is an aggressive subtype of BC, exhibits a high amount of heterogeneity, and frequently displays resistance to chemotherapeutics [3, 4]. TNBCs are associated with younger age at diagnosis (< 40 years) and disproportionately affect African American or Hispanic women [5]. Most often, TNBCs exhibit advanced disease at presentation, a worse prognosis, and due to lack of targeted therapies, they represent a disproportional share of overall BC mortality [3, 4]. There is an urgent unmet need for rationally designed novel therapies that can improve patient response to TNBC treatment and extend overall patient survival.

Proline-, glutamic acid-, and leucine-rich protein 1 (PELP1) is a scaffolding protein with no known enzymatic activity that functions as a proto-oncogene in several cancers including BC [6, 7]. PELP1 expression is an independent prognostic predictor of shorter BC-specific survival and disease-free interval [8]. Recent studies have suggested PELP1 expression has diagnostic utility for metastatic TNBC [9], and PELP1/Ki-67 double high expression in tumors can serve as an independent prognostic factor for predicting TNBC outcomes [10].

Recent studies suggested that PELP1 can function as a coactivator of several nuclear receptors (NRs) inclusive of estrogen receptor alpha (ERα), androgen receptor (AR), glucocorticoid receptor (GR), and transcription factors (TFs) such as E2F1, STATs, and MT53 which are relevant to the progression of TNBC [6, 11]. Additionally, research has shown that PELP1 is crucial for cell cycle advancement [12] and ribosomal biogenesis [13]. Due to its multifaceted activity in TNBC models, PELP1 represents a unique target for therapy.

Previously, a small molecule inhibitor of PELP1 (SMIP34) which displayed a distinct ability to block PELP1 oncogenic signaling was identified and characterized [14]. In this study, we investigated whether SMIP34 has utility in treating TNBC. These results using in vitro models of TNBC showed that SMIP34 is effective in reducing cell viability, reducing invasion, and inducing apoptosis. Mechanistic studies indicated that SMIP34 treatment reduces the levels of PELP1 via proteasomal degradation pathways and alters cell cycle progression. Furthermore, SMIP34 demonstrated effectiveness in reducing proliferation ex vivo and in vivo utilizing cell line derived and patient-derived xenograft tumors.

## Methods

### Cell cultures and reagents

Human TNBC cell lines: BT-549, HCC-1806, HCC-1937, MDA-MB-231, MDA-MB-453, MDA-MB-468 and SUM-159 were obtained from the American Type Culture Collection (ATCC) and were maintained using ATCC recommended media. All model cells utilized were free of mycoplasma contamination. Additionally, STR DNA profiling was used to confirm the identity of cell lines. The GAPDH, p-ERK (42/44), ERK (42/44), p-S6 (s235/236), S6, p-mTOR (S2448), mTOR, p-4EBP1, 4EBP1 and c-Myc antibodies were obtained from Cell Signaling Technology (Beverly, MA). LAS1L, TEX-10, SENP3 antibodies were purchased from Proteintech (Rosemont, IL). The β-Actin antibody (A-2066), WDR18 antibody (HPA050200) and Vinculin antibody (V9264) were purchased from Millipore Sigma (Burlington, MA). The Ki67 antibody (ab1667) was purchased from Abcam (Cambridge, MA). The PELP1 antibody (A300-180A) was purchased form Bethyl Laboratories Inc. (Montgomery, TX).

### Cell viability, colony formation, and apoptosis assays

The effects of SMIP34 treatment on cell viability was assessed by using the MTT cell viability assay in 96-well plates as described [14]. Colony formation assays were done as described [14]. Briefly, BT-549 and MDA-MB-231 model cell lines (500 cells/well) were seeded in 6-well plates and allowed to grow for 5 days in control or SMIP34 treated medium and then medium was changed to normal medium and allowed to grow for 5–7 more days. Cells were then fixed in ice-cold methanol and stained with 0.5% crystal violet solution. The colony area percentage was calculated using NIH ImageJ software. Apoptosis was measured using Annexin V-PI staining (BioLegend, San Diego, CA) according to manufacturer’s protocol.

### Cell invasion assays

The effect of SMIP34 on cell invasion of TNBC cells was determined by using the Corning® BioCoat™ Growth Factor Reduced Matrigel Invasion Chamber assay. MDA-MB-231 and BT-549 cells were treated with vehicle or SMIP34 (20 μM) for 22 h and invaded cells were determined according to manufacturer protocols.

### Cell cycle and RT-qPCR analysis

TNBC cells were treated with either vehicle (0.1% DMSO) or SMIP34 (10 μM) for 48 h. Cells were then trypsinized and harvested in PBS, followed by fixation in ice-cold 70% ethanol for 30 min at 4 °C. Cells were washed again with PBS and stained with a mixture of propidium iodide (PI) and RNase A. The PI-stained cells were subjected to flow cytometry using a BD FACSCalibur™ (BD Biosciences). Total RNA extracted from the TNBC cells was used for real-time PCR. The real-time PCR primers that were utilized to validate the PELP1 target genes were purchased from Millipore Sigma (Burlington, MA).

### Western blotting

TNBC model cells were subjected to cell lysis using either RIPA or NP-40/Triton X-100-lysis buffer containing protease and phosphatase inhibitors followed by Western blot analysis. For proteasome degradation experiments, MG132 was purchased from Sigma (Burlington, MA). After treatment, cells were subjected to cell lysis using NP-40/TritonX-100-lysis buffer containing protease/phosphatase inhibitors and deubiquitinating enzyme inhibitor N-Ethylmaleimide (NEM) (Selleck, Pittsburgh, PA) followed by Western blot analysis.

### Immunohistochemistry (IHC)

IHC was performed as described previously [14]. Briefly, tumor sections were incubated with Ki67 (1:50) or PELP1 (1:150) primary antibody for overnight at 4 °C followed by secondary antibody incubation for 45 min at room temperature. Immunoreactivity was visualized by using the DAB substrate and counterstained with hematoxylin (Vector Lab, Burlingame, CA). A proliferative index was calculated as the percentage of Ki67 positive cells in five randomly selected microscopic fields at 20X per slide.

### Ex vivo tumor studies

Excised tumor tissues from cell line-derived xenograft (CDX) and patient-derived xenograft (PDX) were processed, and cultured ex vivo as previously described [14]. Briefly, tissues were processed and excised into small pieces and cultured on gelatin sponges for 24 h in medium containing 10% FBS. Tissues were treated with vehicle or 20 μM SMIP34 in culture medium for 72 h and fixed in 10% buffered formalin at 4 °C overnight and subsequently processed into paraffin blocks. Sections were then processed for IHC of Ki67 staining.

### In vivo orthotopic tumor models

All animal experiments were performed after obtaining VA and UTHSA IACUC approval. Female 8 weeks-old SCID or NSG mice were purchased from Jackson Laboratory (Bar Harbor, ME). For xenograft tumor assays, model cells (MDA-MB-231, 2 × 10^6^) were mixed with an equal volume of Matrigel and injected into the mammary fat pads of female SCID mice as described [15]. For PDX studies, PDX tumor tissue was dissected into 2 mm^3^ pieces and implanted into the flanks of female NSG mice. When the tumor volume reached ~ 150 mm^3^, mice were randomized for treatment. Based on previous lab data as well as published findings, the numbers of animals needed were chosen to demonstrate differences in tumor incidence or treatment effect. Calculations are based on a model of unpaired data power = 0.8; *p* < 0.05. Once tumors reached measurable size (~ 150–200mm^3^), mice were divided into control and treatment groups (*n* = 7 or 8 tumors per group). The control group received vehicle and the treatment groups received SMIP34 (20 mg/kg/i.p./5 days/week) in 0.3% hydroxypropyl cellulose. Animals were monitored daily for adverse toxic effects. The TM89 and TM96 PDX models were purchased from Jackson Laboratory. Tumor growth was measured by caliper at 3–4 days intervals. At the end of each experiment, animals were euthanized, and the tumors excised, weighed, and processed for IHC staining.

### Statistical analyses

Statistical differences between groups were analyzed with unpaired Student’s t-test and ANOVA using GraphPad Prism 9 software. All the data represented in plots are shown as means ± SE. A value of *p* < 0.05 was considered as statistically significant.

## Results

### SMIP34 treatment reduces cell viability and colony formation in TNBC cells

**Fig. 1:**
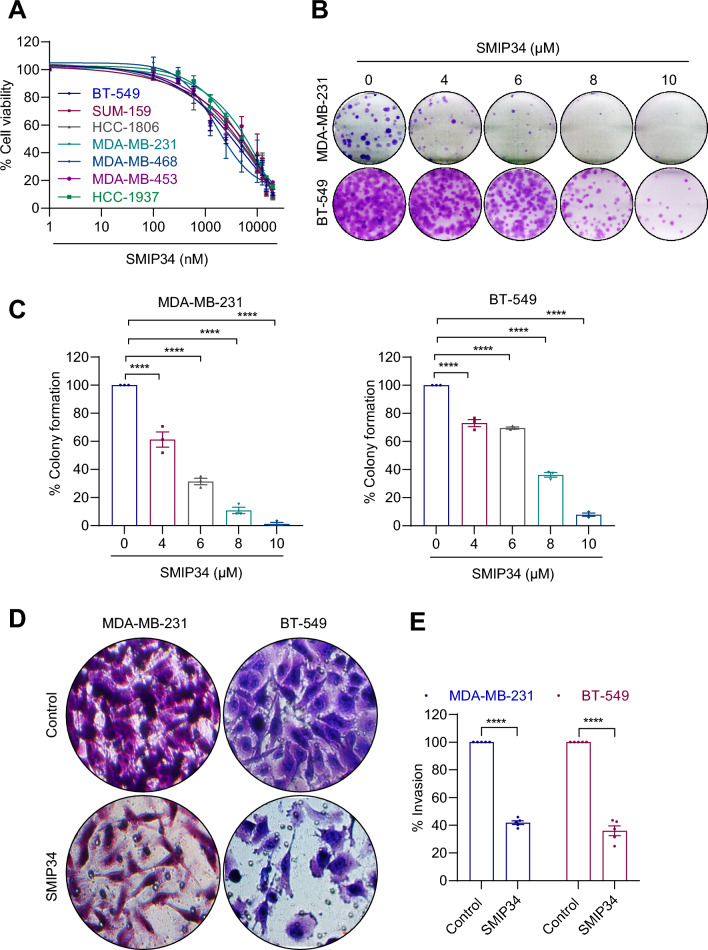
Effect of SMIP34 treatment on in vitro TNBC models. **A**, Effect of increasing doses of SMIP34 on the cell viability of TNBC cells was determined using the MTT cell viability assay (*n* = 3). **B**, Effect of SMIP34 on cell survival was measured using colony formation assays (*n* = 3). Representative images of colonies from each cell type were shown. **C**, Quantification of colonies of MDA-MB-231 and BT-549 cells were shown. **D**, TNBC cells were plated in the upper wells of a BioCoat™ invasion chamber, treated with SMIP34 (20 μM) and invasion was measured at 22 h (*n* = 3). **E**, Quantification of invasion is shown. Data are represented as mean ± SE. ****p < 0.0001

Here, we examined whether SMIP34 exerts growth inhibitory effects in TNBC model cells using cell viability and clonogenic survival assays. In MTT cell viability assays using seven distinct TNBC models, SMIP34 treatment significantly reduced cell viability of TNBC model cells with an IC_50_ ranging from 5 to 10 μM (Fig. 1A). Further, SMIP34 treatment reduced the colony formation ability of MDA-MB-231 and BT-549 cells in a dose-dependent manner (Fig. 1B, C). Published studies have demonstrated that PELP1 promotes invasion of TNBC cells [16]. Therefore, we examined whether SMIP34 treatment reduced the invasiveness of TNBC model cells. Treatment of MDA-MB-231 and BT-549 cells with SIMP34 significantly reduced the invasion of both TNBC model cells compared to controls (Fig. 1D, E).

### SMIP34 treatment promotes apoptosis and alters cell cycle progression

**Fig. 2:**
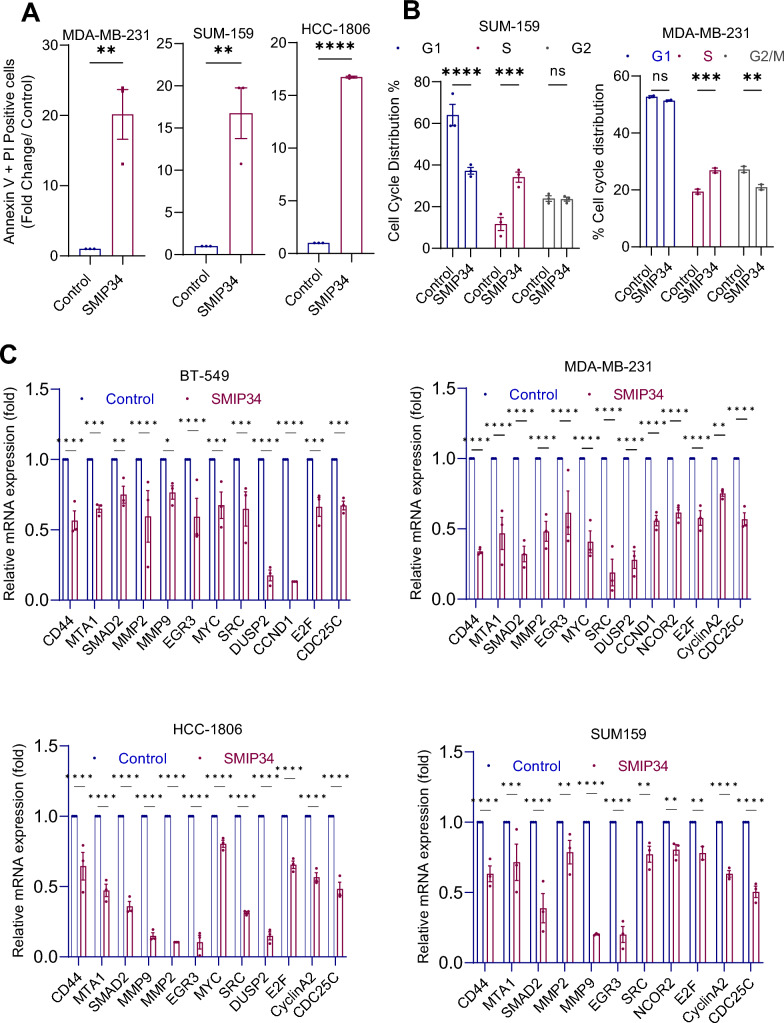
SMIP34 increases apoptosis, alters cell cycle, and decreases downstream PELP1 target genes in TNBC cells. **A**, Effect of SMIP34 (12.5 μM) on apoptosis was measured using Annexin V staining in MDA-MB-231, SUM-159, and HCC-1806 cells. **B**, Treatment with 12.5 μM SMIP34 for 48 h alters cell cycle progression and induces S-phase arrest. **C**, Effect of SMIP34 (12.5 μM) treatment for 24 h on expression of known PELP1 target genes in TNBC model cells was determined by RTqPCR. Data are represented as mean ± SE. *p < 0.05, **p < 0.01, ***p < 0.001, ****p < 0.0001

Next, we evaluated whether SMIP34 treatment promotes apoptosis. MDA-MB-231, SUM-159, and HCC-1806 cells were treated with SMIP34 for 24 h and apoptosis was measured using Annexin V/PI staining assay. Results showed that SMIP34 treatment significantly promoted apoptosis (Fig. 2A). Cell cycle analyses revealed that SMIP34 (12.5 µM) treatment for 48 h promoted S phase arrest in TNBC cell lines (Fig. 2B). RT-qPCR analyses showed that SMIP34 treatment reduced expression of known PELP1 target genes in TNBC (Fig. 2C). Collectively, these data suggest that SMIP34 interferes with cell cycle progression, reduced PELP1-mediated activation of genes and promotes apoptosis.

### SMIP34 degrades PELP1 and reduced PELP1 extranuclear signaling

**Fig. 3:**
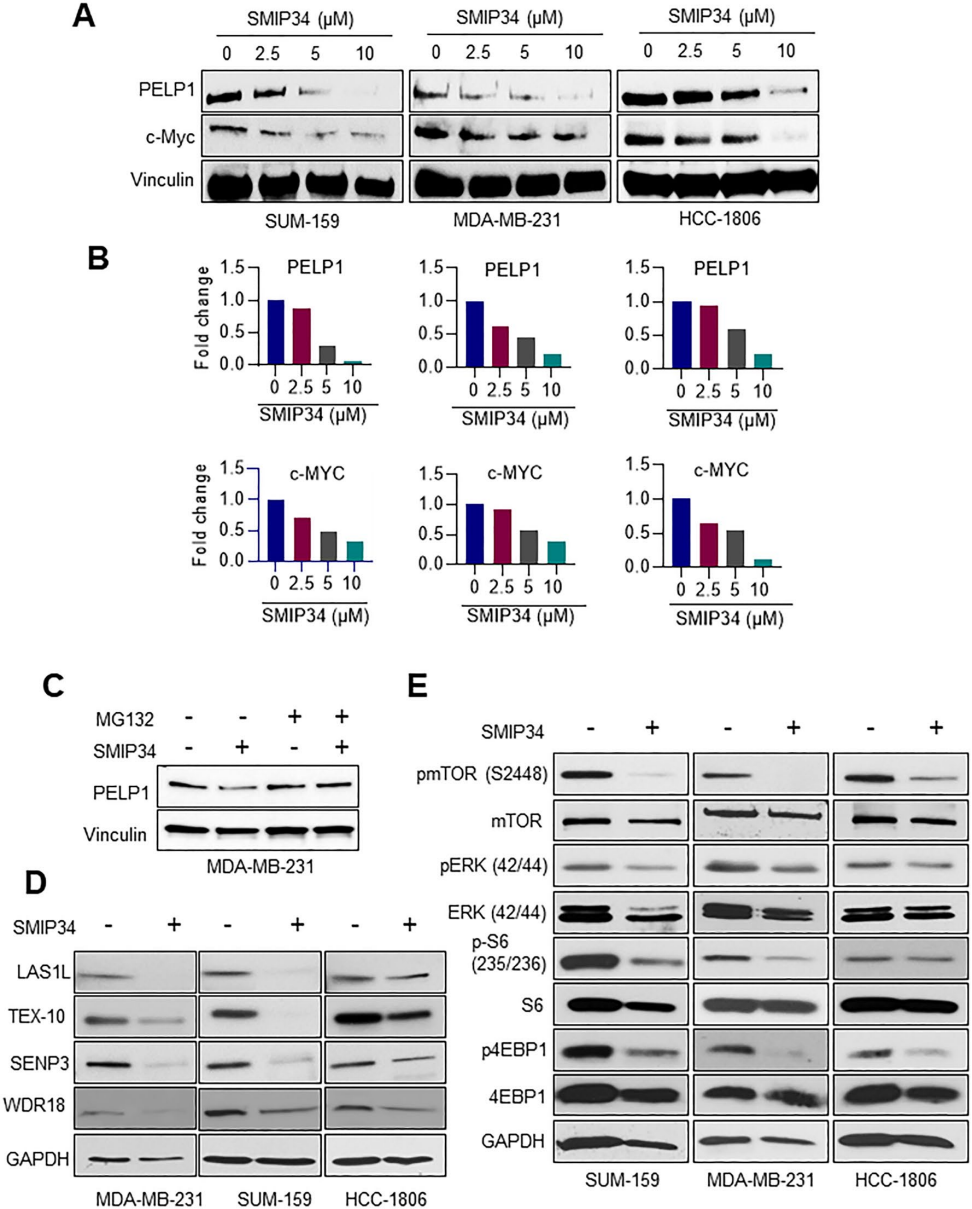
**A,** SUM-159, MDA-MB-231, and HCC-1806 cells were treated with indicated concentrations of SMIP34 for 48 h and expression of PELP1 and cMyc was analyzed by Western blotting. **B**, Western blot quantitation of PELP1 and c-Myc expression in SUM-159, MDA-MB-231, and HCC-1806. **C**, MDA-MB-231 cells were treated with SMIP34 (10 µM) for 24 h in the presence or absence of MG132 (5 µM) and expression of PELP1 was analyzed by Western blotting. **D**, MDA-MB-231, SUM-159, and HCC-1806 cells were treated with SMIP34 (10 µM) and levels of Rix complex proteins LAS1L, TEX-10, SENP3 and WDR18 were analyzed using Western blot analyses. **E**, MDA-MB-231, SUM-159, and HCC-1806 were treated with SMIP34 (10 µM) and status of mTOR, ERK, S6, and 4EBP1 was analyzed using phospho-specific antibodies

Our previously published study suggested that SMIP34 reduced the levels of PELP1 in wild type and mutant ER^+^ BC and therapy-resistant BC cells [14]. We examined whether PELP1 degradation by SMIP34 also occurs in TNBC model cells. Western blot analysis utilizing three different TNBC models confirmed that SMIP34 degrades PELP1 and reduced cMyc expression in a dose-dependent manner (Fig. 3A, B). SMIP34 mediated degradation of PELP1 was blocked by the addition of MG132, a proteasomal inhibitor (Fig. 3C). By being a component of the Rix complex (PELP1-TEX10-SENP3-WDR18), PELP1 plays a crucial function in ribosome biogenesis [17, 18] and has also been demonstrated to operate as a regulatory point for mammalian 60S maturation [19]. Therefore, we examined whether SMIP34 mediated downregulation of PELP1 contributes to destabilization and downregulation of Rix complex proteins. Western blot analyses of total lysates of SMIP34 treated TNBC models showed significant downregulation Rix complex proteins including LAS1L, TEX-10, SENP3 and WDR18 (Fig. 3D). In addition to PELP1 genomic functions, PELP1 is also known to play a role in the extranuclear signaling via direct interactions with Src-ERK and mTOR kinases [6]. Western blot analysis of TNBC cell lysates treated with SMIP34 demonstrated that SMIP34 treatment substantially lowers PELP1 downstream signaling including phosphorylation of mTOR, ERK, pS6, and p4EBP1 compared to control (Fig. 3E).

### SMIP34 is effective in reducing the proliferation of breast tumor explants

**Fig. 4:**
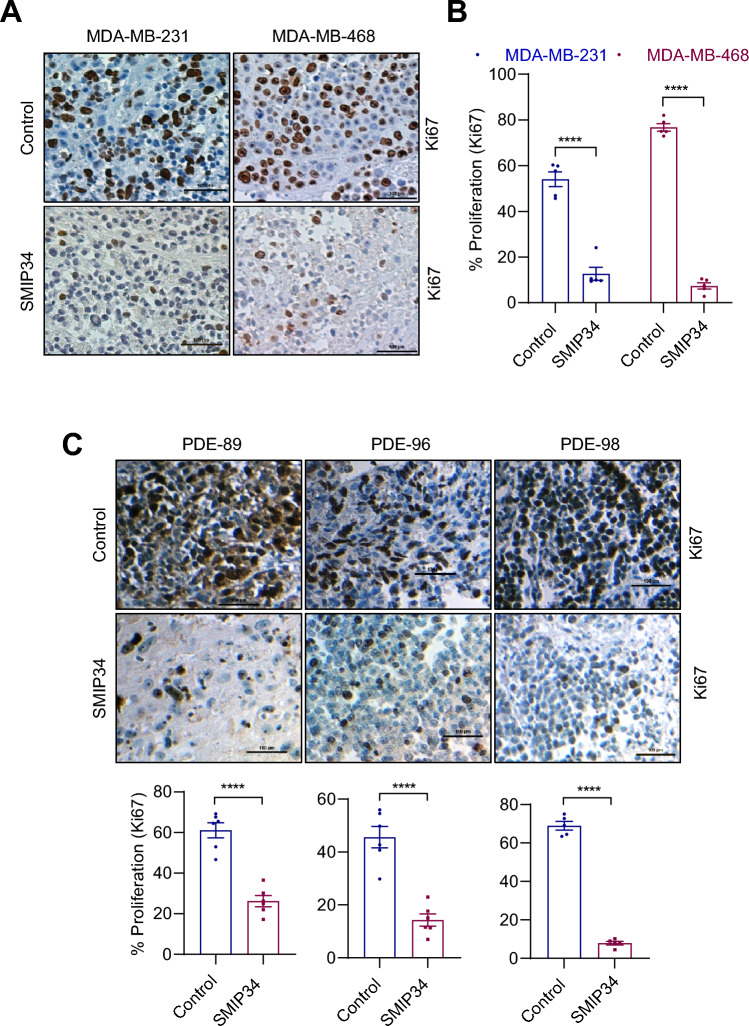
SMIP34 inhibits the growth of TNBC breast tumor explants. **A**, TNBC CDX tumor explants were treated with SMIP34 (20 μM) for 72 h, and the proliferation was determined using Ki67 immunostaining. Representative Ki67 staining from one tumor treated with vehicle or SMIP34 is shown. **B**, Quantitation of Ki67 immunostaining is shown (*n* = 3). **C**, TNBC PDX explants were treated with SMIP34 (20 μM) for 72 h and the proliferation was determined using Ki67 immunostaining. Representative Ki67 staining from one tumor treated with vehicle or SMIP34 is shown. The Ki67 expression in explants (*n* = 3) is quantitated. Data are represented as mean ± SE. ****p < 0.0001

The efficacy of SMIP34 was analyzed utilizing explant assays [20]. Explant tests enable the assessment of SMIP34's efficacy on TNBC tumors while preserving the original tumor tissue architecture. For this assay, tumor tissues derived from both CDX and PDX tumors were used. Results showed that SMIP34 treatment is effective in reducing the proliferation of TNBC CDX tumors (Fig. 4A, B). Further, treatment with SMIP34 also reduced the proliferation of TNBC PDX tumors (Fig. 4C). Collectively, these results suggest that SMIP34 is effective in reducing the proliferation of TNBC tumors ex vivo.

### SMIP34 reduced TNBC tumor growth in vivo

**Fig. 5:**
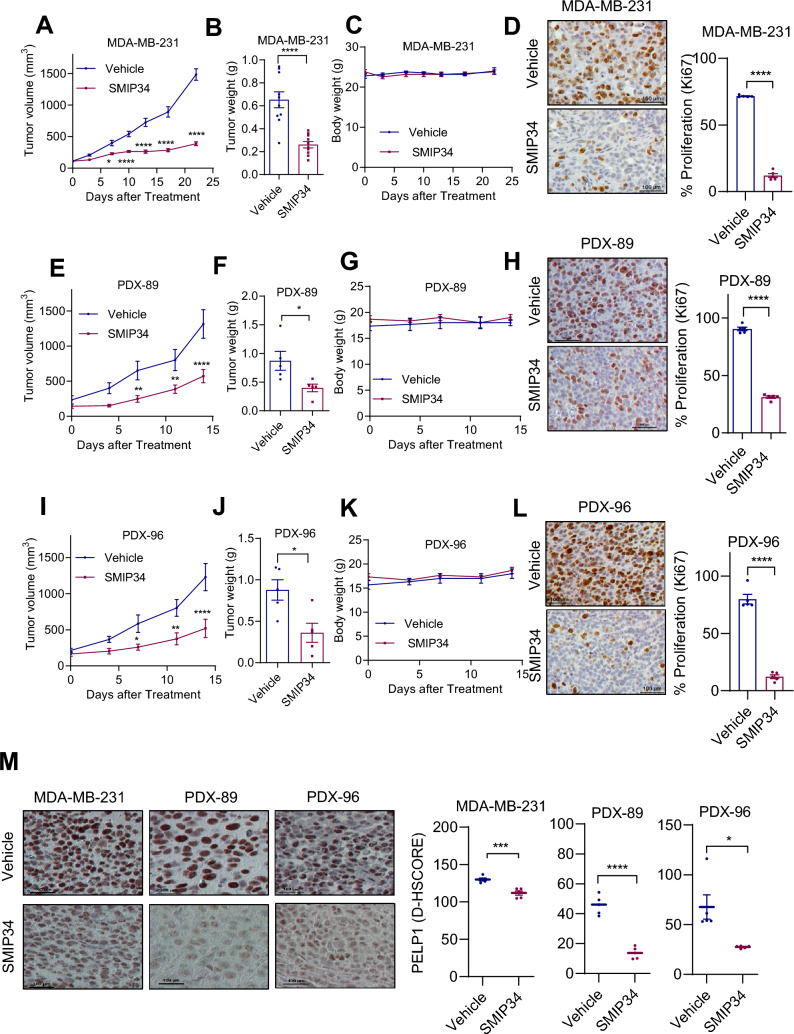
SMIP34 inhibits the growth of CDX and PDX tumors in vivo*.* **A**–**C**, MDA-MB-231 xenografts (*n* = 10 tumors/group) were treated with vehicle or SMIP34 (20 mg/kg/i.p./5 days/week). Tumor volumes are shown in the graph. Tumor weights (**B)** and body weights (**C**) of vehicle and SMIP34 treated animals are shown. **D**, Representative Ki67 staining of xenograft tumors from MDA-MB-231 treated with vehicle or SMIP34 is shown. **E–G**, PDX-89 xenografts (*n* = 6 tumors/group) were treated with vehicle or SMIP34 (20 mg/kg/i.p./5 days/week). Tumor volumes are shown in the graph (**E)**. Tumor weights (**F**) and body weights (**G**) of vehicle and SMIP34 treated animals are shown. **H,** Representative Ki67 staining of xenograft tumors from PDX-89 treated with vehicle or SMIP34 is shown. **I-K**, PDX-96 xenograft tumors (*n* = 6 tumors/group) were treated with vehicle or SMIP34 (20 mg/kg/i.p./5 days/week). Tumor volumes are shown in the graph (I). Tumor weights (**J**) and body weights (**K**) of vehicle and SMIP34 treated animals are shown. **L**, Representative Ki67 staining of xenograft tumors from PDX-96 treated with vehicle or SMIP34 is shown. **M,** PELP1 expression was analyzed by IHC and quantitated (*n* = 5). Data are represented as mean ± SE. *p < 0.1; **p < 0.01; ***p < 0.001; ****p < 0.0001

To examine the efficacy of SMIP34 on TNBC progression in vivo, MDA-MB-231 TNBC cells were utilized in a xenograft assay. Xenografts were established by injecting 2 × 10^6^ cells in the mammary fat pad of female SCID mice. Mice with MDA-MB-231 xenografts (*n* = 10) were randomized and treated with SMP34 (20 mg/kg/i.p./5 days/week). SMIP34 treatment significantly reduced the tumor progression (Fig. 5A) and tumor weight (Fig. 5B) compared to vehicle. SMIP34 treatment showed no significant effect on body weights of mice (Fig. 5C). To enhance translatability, the utility of SMIP34 using two distinct TNBC PDX models. PDX tumors were established by implanting tumor pieces (2 mm^3^) in the mammary fat pad of female mice as described in the methods section. When PDX tumors (*n* = 6) reached ~ 200 mm^3^, mice were randomized and treated with SMIP34 was evaluated  (20 mg/kg/i.p./5 days/week). Results showed that SMIP34 is efficient in reducing the growth of PDX tumors (Fig. 5E, I) and tumor weights (Fig. 5F, J) compared to vehicle. Mice body weights in the vehicle and SMIP34 treated groups remain unchanged (Fig. 5G, K). SMIP34 treated CDX and PDX tumors showed reduced proliferation (Ki67 staining) compared to vehicle treated group (Fig. 5D, H, L). Further, SMIP34 treated tumors exhibited reduced levels of PELP1 (Fig. 5M). Collectively, these results suggest that SMIP34 is effective in reducing the progression of TNBC tumors in vivo.

## Discussion

TNBC lack targeted therapies, hence creating efficient therapeutic alternatives for TNBC constitutes an unmet need in disease management. A small molecule inhibitor, SMIP34, that blocks the oncogenic activities of PELP1 has been discovered in our previously published study which shows that SMIP34 blocks the oncogenic activities of PELP1 in ER + BC [14]. Since PELP1 signaling has been linked to TNBC progression in several studies [6, 16, 21], in this study we investigated whether recently identified PELP1 inhibitor SMIP34 may be used as a therapeutic agent for TNBC. Results showed that SMIP34 reduces cell viability, colony formation, and cell invasion of TNBC models. Additionally, SMIP34 therapy markedly reduced PELP1 levels and enhanced the apoptosis of TNBC model cells. The results from this study demonstrated the in vitro, ex vivo, and in vivo efficacy of SMIP34.

PELP1 signaling is shown to play a critical role in the cancer progression via its ability to activate multiple signaling pathways including hormonal signaling [22], cell cycle progression [12], ribosomal biogenesis [13], and DNA damage response [7, 23]. Targeting of PELP1 enhances the chemotherapeutic response of TNBC via inhibition of cell cycle progression and activation of apoptosis [21]. In fact, published studies utilizing knock down approaches [24], blockage of PELP1 downstream signaling utilizing a KDM1A inhibitor [24], and reducing PELP1 expression using a CDK inhibitor Roscovitine [25], demonstrate the benefit of targeting PELP1 in reducing cancer progression. The lack of an inhibitor that directly binds and blocks PELP1 signaling represents a knowledge gap in clinical translation of these findings. The results from this study demonstrated that SMIP34 has ability to reduce the levels of PELP1 in TNBC models.

Published research demonstrated that the PELP1-TEX10-WDR18 complex regulates ribosome biogenesis, and PELP1 control the rate of ribosome synthesis [17]. The nucleolus is where PELP1 localizes and contributes to the efficient synthesis of 28S rRNA [18]. Recent research employing global analysis and CRISPR PELP1 knockout model cells demonstrated that PELP1-mediated oncogenic capabilities include regulation of ribosome biosynthesis [26]. According to the recently published Cryo-EM structure of the WDR18/PELP1 Rix1 complex (PDB code 7UWF) [27], the PELP1 homodimer functions as the assembly's central component. Our findings from the current study indicate that SMIP34 destabilizes the Rix1 complex proteins, including WDR18, TEX10, LASIL, and SENP3, and that this causes a significant drop in the amounts of these proteins.

PELP1 is a prognostic indicator of poorer BC survival [8] and its overexpression contributes to BC therapy resistance [11, 24] and metastases [28]. Previous studies found that treatment of xenograft tumors with PELP1-siRNA liposomes significantly reduced tumor volume [24]. Furthermore, PELP1 knockdown reduced the in vivo metastases of TNBC [16] and PELP1-targeted therapies enhanced the response to chemotherapies [21]. In this study, results demonstrated that SMIP34 has efficacy in reducing the cell viability in seven distinct TNBC cell lines. Furthermore, studies using TNBC tumors ex vivo and CDX and PDX tumors in vivo demonstrated that SMIP34 has utility in reducing the progression of TNBC tumors, and these results are in accordance with published studies on the oncogenic role of PELP1 in TNBC.

In TNBC, PELP1 expression is a recognized independent prognostic marker for indicating a worse prognosis [10]. When compared to node-negative specimens, PELP1 expression is more prominent in invasive breast cancers and metastatic tumors [29]. Expression of PELP1 is linked to several genes involved in the epithelial mesenchymal transition (EMT) and that influence metastasis [28]. The results from this study are in concordance with published findings and demonstrate that SMIP34 is effective in reducing invasiveness in TNBC model cells. Further, our results indicated that SMIP34 administration enhanced apoptosis in these cell lines. Future research utilizing global gene expression analysis is required to fully comprehend the molecular processes by which SMIP34 therapy inhibited the growth of TNBC models.

In conclusion, our findings show that SMIP34 decreases TNBC cell growth in vitro, ex vivo, and in vivo via degradation of PELP1. These findings demonstrate that SMIP34 is a new agent for therapeutic intervention of TNBC that targets PELP1 oncogenic signaling.

## Data Availability

The datasets analyzed are available from the corresponding author on reasonable request.
